# Patients’ Opinions on the Quality of Services in Hospital Wards in Poland

**DOI:** 10.3390/ijerph20010412

**Published:** 2022-12-27

**Authors:** Mariola Borowska, Urszula Religioni, Anna Augustynowicz

**Affiliations:** 1 Department of Economics of Health and Medical Law, Medical University of Warsaw, 02-091 Warsaw, Poland; 2 School of Public Health, Centre of Postgraduate Medical Education of Warsaw, 01-813 Warsaw, Poland

**Keywords:** health care quality, patient, patient experience, patient-centeredness

## Abstract

Introduction: Patient opinion surveys have become a widely used method for assessing key aspects of the functioning of medical facilities and, thus, of the functioning of the entire health care system. They are a prerequisite for developing patient-centered care and an essential component of quality improvement programs. In many countries, including Poland, patient opinion surveys are written into the accreditation standards of medical institutions. Patient’s readiness to recommend a hospital is a recognized indicator of the quality of patient-centered care. In a report on strategies for improving the quality of health care in Europe published in 2019 by WHO and the OECD (Organisation for Economic Cooperation and Development), patient’s readiness to recommend a hospital was cited as one of the basic indicators of ‘patient centeredness’ along with patient satisfaction. Therefore, as well consideration of the quality of medical care, a patient recommendation index was also used in the study presented in this paper. The index was based on the answers to questions about the patient’s readiness to recommend a hospital ward to family and friends. Aim: The aim of the study was to investigate patients’ opinions on the quality of services in particular hospital wards. A patient opinion survey can be used to improve the quality of services and monitor the effects of health-related activities, identify areas that need improvement, motivate medical staff and prevent their burnout, build a trusting relationship with patients, and compare the quality of health care in various facilities. Material and methods: The study was carried out in March 2022. The patient opinion survey was conducted using the CAWI (Computer-Assisted Web Interview). The sample selection was purposive. The respondents were patients with a history of hospitalization. The sample selection used an algorithm for the random selection of patients who met the criteria for the sample. The inclusion criterion was hospitalization in the 12 months prior to the study. A standardized questionnaire was used that was aimed at the assessment of the quality of medical care and the patient’s rights to information. Additionally, the survey contained questions about the demographic characteristics of the respondents. Results: A total of 38% of patients with a history of hospitalization expressed criticisms. The majority of statistically significant differences were observed when differentiating respondents according to age. Elderly persons significantly more often declared having been treated with respect and interest. They also rated more highly the meals served in the hospital, effective pain treatment, and respect for the patient’s dignity and intimacy during diagnosis and treatment. Younger persons assessed all these aspects of hospitalization less favorably. Conclusions: Variables including age and the level of income had a statistically significant influence on the opinion of the respondents. Elderly persons assessed most aspects of the quality of care in a hospital ward more favorably. There were a similar number of “promoters” (36%) and “detractors” (38%) of the quality of hospital services. Detractors mainly pointed to long waiting times for hospital admission, the poor quality of medical and nursing care, and unappealing meals. The promoters emphasized the high quality of medical and nursing care and the favorable conditions of the accommodation. Regular patient satisfaction surveys are helpful in identifying areas in which the functioning of a medical entity requires changes.

## 1. Introduction

The quality of medical services is one of the key attributes of the provision of health care. Increasingly effective methods for the management of healthcare facilities are required due to dynamic changes in the provision of medical services and increased patient awareness [1]. Continuous improvement in medical services and their adaptation to patients’ needs are the most important issues for health care today [2].

Quality is a very broad concept. With respect to health care, it should be defined not only from the perspective of treatment results, but also with consideration of the conditions in which the treatment process occurs, the atmosphere in which health services are provided to patients and the cost-result relationship. All these factors translate into quality [3]. Quality in health care is essential, not only for the functioning of medical facilities as such, but, most of all, for the health and comfort of patients [4]. According to the World Health Organization, quality involves the result (technical quality), the use of resources (economic efficiency), the organization of services and patient satisfaction [5,6]. Quality in health care is defined not only according to material criteria, but also according to sociological and psychological criteria and concepts. Quality of care means:

- application of all the achievements of modern medicine that are necessary to satisfy patients’ needs,
- health care that is measurable, acceptable, holistic, continuous and documented,
- health care that meets the relevant criteria of care,
- effective activities that raise the level of health and satisfaction of the population, consistent with the appropriate use of resources for providing care to the community and individuals,
- and is a multidimensional and comprehensive concept [7,8,9].

Patient opinion surveys have become an increasingly popular method used to assess key aspects of the functioning of medical facilities and, thus, the entire health care system. It is a prerequisite for developing patient-centered care (patient-centeredness) and an essential component of quality improvement programs. In many countries, including Poland, their use is also written into the accreditation standards of medical facilities [10,11]. Patient’s readiness to recommend a hospital is an important indicator of the quality of treatment in patient-centered health care. In a report on strategies to improve the quality of health care in Europe, which was published by WHO and the OECD in 2019, patient’s readiness to recommend a hospital was cited as one of the basic indicators of ‘patient centeredness’ along with patient satisfaction [12]. Therefore, alongside the quality of medical care, a patient recommendation index was also measured in the study presented in this paper. It was based on the answers to questions about patients’ readiness to recommend a hospital ward to family and friends. The NPS (net promoter score) methodology was developed in 2003 by Frederik F. Reichheld and described in an article in the Harvard Business Review [13]. Since then, it has become very popular, replacing previously used complicated customer satisfaction questionnaires. Respondents are asked: “Would you recommend our hospital ward to family and friends if they needed similar care or therapy?” [14,15,16]. A follow-up question sought to find out why the patient marked a specific value on the scale, which provided many valuable opinions about the quality of medical care and the management of the medical entity.

Accreditation standards define the manner of providing health care and have a positive impact on the awareness of medical professionals and managers of health care facilities. Quality improvement and patient safety are some of the requirements that must be met by a hospital to be given accreditation. Health care quality improvement means continuous monitoring, analysis and improvement of treatment and management. Quality requires strong leadership, good work organization, cooperation of hospital staff, and effective assessment of quality level. Quality improvement is aimed at reducing risk in a group of patients and caregivers. It involves monitoring and evaluation of quality indicators, on the basis of which specific improvement methods are implemented. The level of improvement is conditioned by the knowledge of the current functioning of a facility, the definition of progress expected and the time necessary to make improvements (quality monitoring). In light of the above, it is necessary to assess the functioning of a facility, i.e., to collect relevant data according to reliable methodologies, to evaluate and analyze data, to identify changes that should be introduced to ensure quality improvement, to implement changes and to undertake further evaluation to determine whether the introduced changes translate into improvement. Through evaluation of patient satisfaction and the quality of medical care, a medical facility can meet accreditation standards and, even more importantly, respond to the changing needs of patients and adapt to the environment in which it operates [10,17,18,19].

The aim of the study presented in this paper was to examine the impact of patients’ opinions on the quality of services in hospital departments on the identification of areas for improvement in the operation of the healthcare entity. The quality of care defined by patients serves to identify specific areas for improvement (the hospital as an organization that learns), to motivate medical staff and prevent their professional burnout, to build a relationship of trust with patients (if the hospital chooses to publish critical comments and inform the public about corrective actions taken), to compare the quality of care in different facilities (in departments with similar characteristics) to offer patients a wider choice, and to motivate facilities to continuously improve the quality of care.

## 2. Materials and Methods

The study was carried out in March 2022. The patient opinion survey was conducted using the CAWI. The sample selection was purposive. The respondents were patients with a history of hospitalization. The sample selection was supported using an algorithm for the random selection of patients who met the criteria of the sample. The inclusion criterion was hospitalization in the 12 months prior to the study.

A standardized survey was used that contained questions on the assessment of the quality of medical care, including questions about the respect shown by medical staff to patients, the quality of meals served, hygiene-related issues, such as washing and disinfection of hands by medical staff before approaching the patient, the effectiveness of pain treatment, hospital conditions, and patients’ opinions on whether they had achieved the best possible treatment results during hospitalization. The second part of the survey concerned patients’ rights to information. It examined whether medical staff listened to the patient, informed the patient about their health in an understandable way, informed them about possible treatment methods and their consequences, and asked the patient for their opinion when choosing treatment methods. In addition, the survey contained questions about patient demographic characteristics, such as age and sex, place of residence (number of inhabitants and voivodship), education, household situation, job position and income level.

The factors affecting the quality of medical care, general assessment of the hospital/hospital ward and the likelihood of recommending it to patient’s family and friends were examined. A net promoter score (NPS) was used to check how likely the clients/patients were to recommend a product or service to their family and friends. Depending on their answer to the question on a scale of 0–10, the respondents were divided into three groups: promoters, neutral respondents and detractors. Respondents who gave 9–10 points on the scale were defined as promoters: people who were satisfied and likely to recommend the service; 7–8 points were given by those defined as neutral respondents: patients who were satisfied, but not eager to promote the service; 0–6 points were given by those defined as detractors: dissatisfied patients who did not recommend a service, and who might even discourage others from using the service due to its poor quality. The NPS index is widely used in research on the quality of services, including medical services. Importantly, to obtain a full picture of the quality of a given service, apart from response to questions with a score of 0–10, the respondents were asked a further qualitative open question to justify the number of points given. The respondents were asked about the reason for providing a specific score on the scale of 0–10 when assessing the hospital that they stayed in.

The following aspects of the quality of medical care were analyzed: treatment of patients by medical staff with care and respect, the quality and freshness of meals, hygiene issues, such as washing and disinfection of hands by medical staff before approaching the patient, effectiveness of pain treatment, hospital conditions, including the size of rooms and ensuring privacy and dignity, as well as patients’ opinions on achievement of the best possible treatment results during hospitalization. The most statistically significant differences were observed when differentiating respondents based on the net promoter score (NPS). For these data, statistically significant differences were observed for each group: promoters, neutral respondents and detractors.

Treating patients with respect and attention, the quality and freshness of served meals, hygiene issues, such as washing and disinfecting hands by medical staff before approaching the patient, and the effectiveness of pain treatment were assessed on a scale: never, sometimes, usually, always. Hospital conditions, including the size of the rooms and ensuring patients privacy and dignity, and patients’ opinions on the achievement of the best possible treatment result during their hospital stay were assessed on a scale: definitely not, rather not, rather yes, definitely yes.

The results show only statistically significant values. The standard confidence level was 95%. The significance level refers to the percentage as well as the sample size, which in this case means significance versus total. Parametric tests were used to verify the hypotheses regarding the values of proportions in the general population or to compare the value of proportions in several populations, based on the knowledge of the value of this proportion in a random sample in a population (or two or more samples).

## 3. Results

The study sample consisted of 801 persons, 57% of whom were women and 43% men. The majority of respondents were patients aged 25–34 (28%) and those aged 35–44 (22%). Persons aged 55–64 accounted for 11% of the research sample and people over 65 years of age accounted for 10%. A total of 44% of the respondents had undergone higher education, 59% women and 41% men. A secondary school level of education was reported by 43% of the respondents, of which 55% were women and 45% men. A primary school level of education was reported by 13% of the respondents, 53% of whom were women and 47% men. The majority of respondents were inhabitants of rural areas (21%), and towns with over 500,000 inhabitants (17%). With respect to job position, working persons accounted for 69% and non-working persons accounted for 31% of the study sample. An average net income of up to PLN 3000 was reported by 37% of the respondents and of over PLN 5000 was reported by 20% of the respondents. A total of 12% of the respondents refused to answer the question about salary.

Firstly, we asked about patients’ recommendations of a hospital/hospital ward to family and friends (Table 1).

A total of 36% of the sample were promoters, 27% were neutral respondents, and 38% were detractors. Detractors mainly pointed to long waiting times for admission to hospital, poor quality of medical and nursing care and unappealing meals. Some also referred to other important factors that could affect the quality of care, such as shortage of medical staff, and the overly heavy workload of medical personnel. The study was carried out during the pandemic, so some patients also referred to not being able to be visited by family. The promoters highlighted the high quality of medical and nursing care and good conditions of the accommodation.

The patients’ opinions on being treated with due interest and care by the medical staff in terms of respecting patient rights and involving the patient in the treatment process are very important (Table 2). 

Detractors (NPS scale) declared significantly less frequently (10%) that, during their stay in hospital, medical staff always treated them with due interest and care. The answer ‘sometimes’ was given significantly more often (53%). Promoters (NPS scale) declared significantly more often (65%) that, during their hospital stay, medical staff treated them with due interest and care. Considering the demographic data, the greatest number of statistically significant differences was observed when differentiating respondents according to age. The highest values of differences concerned the answer ‘always’ among the oldest patients. Those aged 55–64 and over 65 years reported 48% and 50% more often, respectively, that the hospital medical staff always treated them with due interest and care. The respondents aged 18–24 years reported the same answer (‘always’) 17% less often. The answers of the respondents were also analyzed with respect to their net monthly income. Statistically significant differences were observed for persons with an income of up to PLN 3000 net (the answer ‘sometimes’ was indicated 21% less often) and for those with a net income of PLN 3001–5000 (the answer ‘sometimes’ was indicated 33% more often).

We investigated a further important aspect—the quality and conditions of serving meals in hospitals, which have a direct impact on patients’ health, recovery and, in the case of inadequate conditions, the spread of infectious diseases in the ward (Table 3).

Detractors (NPS scale) declared significantly more often that they never (33%) and sometimes (37%) were given tasty, fresh and hygienically served meals during their stay in hospital. However, promoters (NPS scale) declared significantly more often that they always (43%) received tasty, fresh and hygienically served meals during their hospital stay. With respect to age, statistically significant differences were observed mainly in in the groups of young respondents (18–24 and 25–34 years of age). A total of 37% of those aged 18–24 years indicated that they were only sometimes given tasty and fresh meals, and only 14% of this group declared that the meals were always of adequate quality and served hygienically. Patients aged 25–34 significantly more often (21%) indicated that their meals were never tasty, fresh or hygienically served.

A further important aspect of medical care is hygiene compliance among medical personnel, which affects the epidemiological safety of patients (Table 4).

Detractors (NPS scale) significantly more often declared that the hospital staff never (14%), or sometimes (31%) washed or disinfected their hands before approaching the patient. Promoters (NPS scale) declared significantly more often that the staff always (67%) washed or disinfected their hands before approaching them. With respect to age, statistically significant differences were observed in the group aged 55–64 years. Respondents in this age group significantly less often indicated that the medical personnel only sometimes (13%) washed or disinfected their hands.

We asked how often pain treatment in hospital was effective in connection with patients’ quality of life and well-being (Table 5).

Detractors (NPS scale) significantly more often reported the answer that the pain was never (8%) or sometimes (36%) successfully treated during their hospitalization. Promoters (NPS scale) declared significantly more often that the pain was always (64%) treated effectively during their stay in hospital. With respect to age, statistically significant differences were observed mainly in the groups aged 25–34 years and 55–64 years. Persons aged 25–34 significantly less often indicated that the pain was always (31%) successfully treated. However, patients aged 55–64 years significantly more often indicated that the pain was always (53%) successfully treated during their hospitalization.

We undertook an analysis of patients’ opinions on hospital conditions and the accessibility of diagnostics and treatment with respect to patient dignity and intimacy, which is very important in relation to patients’ rights (Table 6).

Detractors (NPS scale) gave a ‘definitely not’ answer (19%) and a ‘rather not’ answer (37%) significantly more often. Promoters (NPS scale) declared ‘definitely yes’ significantly more often (51%). With respect to age, statistically significant differences were observed mainly in the age group 55–64 years. Persons aged 55–64 significantly more often indicated the answer ‘definitely yes’ (38%).

Subjective analysis of patients’ opinions on achieving the best possible treatment outcome during hospitalization, indicates overall patient satisfaction in terms of value-based healthcare (Table 7).

Detractors (NPS scale) significantly more often gave the answers ‘definitely not’ (18%) and rather not’ (34%). In contrast, the answer ‘rather yes’ was indicated significantly less often by detractors (42%). Promoters (NPS scale) chose the answer ‘definitely yes’ significantly more often (52%). With respect to age, statistically significant differences were observed mainly in the age group 18–24 years. Persons aged 18–24 years significantly more often gave the answer ‘definitely not’ (25%) and significantly less often chose the answer ‘definitely yes’ (15%). The respondents’ answers were also analyzed in terms of achieved monthly net income. Statistically significant differences were observed for persons with an income of more than PLN 5000 net (the answer ‘definitely yes’ was reported 37% more often).

## 4. Discussion

Patients’ readiness to recommend a hospital is a recognized indicator of the quality of treatment in patient-centered health care. In a report on strategies to improve the quality of health care in Europe, which was published by WHO and the OECD in 2019, patients’ readiness to recommend a hospital was one of the basic indicators of ‘patient centeredness’, as well as patient satisfaction [12]. Therefore, the study presented in this paper, in addition to assessing the quality of medical care, also used a patient recommendation index, which was based on answers to a question about the readiness to recommend a hospital ward to family and friends. Respondents defined in the first question as detractors negatively assessed particular aspects of the quality of health care much more often. An equally important (though considered by some researchers to be more important) open-ended question was used, enabling respondents to ‘fill in the gaps’, i.e., providing an opportunity to gain feedback from dissatisfied patients on the reasons for their dissatisfaction, in order to use the information to improve current processes and make necessary changes to improve the quality of changes [20,21]. Many approaches to measuring patient satisfaction are reported in the literature. Due to the use of different measurement methods, the results obtained cannot always be straightforwardly compared. In 2012, the British National Health Service (NHS) introduced an obligation to examine patients’ opinions using a method based on a modified version of the NPS, the so-called “Friends and Family Test” (FFT). The question asked in this test is “How likely are you to recommend our ward to friends and family if they needed similar care or treatment?” [14,15,22]. In a comparative study on different methods of measuring patient satisfaction that was conducted in six hospitals in the Netherlands, the authors changed the method of calculating the indicator, and defined detractors as respondents who gave answers 0–5, neutral respondents as those who gave answers 6–7, and promoters as those who gave answers 8–10. This change resulted from the necessity to adjust the interpretation of the scale to the cultural context. In the Netherlands, there is a school grading scale of 1 to 10, with 8 being considered a very good grade and 6 as the pass threshold [16]. By including a follow-up question to explore why the patient marked a specific value on the scale, many valuable patients’ opinions were obtained, which concerned not only the quality of medical care, but also the management of the medical entity.

In the study, respondents categorized as detractors reported long waiting times for hospital admission, poor quality of medical and nursing care, and unappealing meals. Some detractors also highlighted extremely important factors that could contribute to the poor quality of care, i.e., shortages of medical staff and the excessive workload of medical staff. However, respondents categorized as promoters reported a high quality of medical and nursing care as well as good conditions in their accommodation. The assessment of the impact of the COVID-19 pandemic on the level of stress among the nursing staff changed in comparison to the pre-pandemic period, which was reflected in an increase in stress symptoms. Aggravating factors reported included the fear of transmitting the virus from the workplace to relatives, the fear of a threat to one’s own life and health and that of relatives, rapid organizational changes, and continuous work under an increased sanitary regime. All these factors translated into a heavy workload, high levels of stress and a high risk of burnout among the nursing staff [23]. A study conducted on a group of patients of the Autonomous Public Teaching Hospital No. 4 in Lublin showed high and medium levels of patient satisfaction with nursing care, in terms of the accommodation that was provided and the help given by the nursing staff, the cleanliness and aesthetics of the hospital/ward rooms, necessary assistance during washing or bathing, conditions for rest and sleep, and the provision of assistance in getting up, sitting and walking. Patients’ satisfaction with nursing care was low in terms of assistance in airing rooms, meeting physiological needs, free time management and physical exercise and rehabilitation [24]. The attitude of nurses, their respect for the patients’ dignity, and assistance in everyday activities have a decisive impact on the level of patients’ satisfaction with a stay in a hospital ward. From the patients’ perspective, these are the most important aspects for assessing a hospital. The study presented in this paper shows that nursing care was rated relatively highly. Patients aged 55–64 and 65+ years assessed the medical staff’s respect for the patient much more highly than those of younger age. Other research conducted among primary care patients has confirmed that interpersonal aspects are very important to patients. Proper communication is especially important for patients when usual visual cues are missing. Communication emerged as one of the highest-rated aspects of care when patient satisfaction with online consultations was assessed. The most highly rated variables were those of empathy and respect for patients; patients appreciated being treated by doctors with care, respect and patience. Patients were also found to report high levels of satisfaction with the comprehensiveness of care, indicating that the primary health care units were able to meet most of the patients’ health needs [25]. In a study of patient satisfaction with services provided in inpatient health care in the Kujawsko–Pomorskie Voivodship, the pro-patient approach of the nursing staff was rated very positively or positively by 90% of respondents. The carefulness of performing procedures by nurses was assessed very positively and positively by 90% of respondents. The availability of nursing staff during the day was poorly assessed by only 0.4% of the respondents. The availability of nursing staff at night was rated badly and very badly by 3.5% of respondents [26]. Similar results were obtained in another study on the level of patient satisfaction, where patients aged 65+ rated the quality and availability of nursing care much more highly [27]. Another study on patient satisfaction conducted by Sierpińska and Dziuba showed that 87% of respondents rated nurses’ friendliness as very good and the response time to patients’ requests as very good [28]. The provision of information on treatment procedures and post-operative management was assessed as very good by 70% of respondents. Hygiene assistance was rated very positively by 76% of the respondents. Similar results were also obtained in another study aimed at the assessment of medical care, in which 53% of patients rated nursing care as very good, 43% as good, and 1% as bad [29]. Another study showed that providing information on the purpose and types of nursing procedures was rated positively by 78% of patients. Opinions on the attitude of nurses and their professionalism were also very positive: 84% of respondents positively assessed the level of professionalism, and over 78% of patients appreciated the nursing staff’s friendliness and understanding [30,31].

Statistically significant differences were observed with respect to the quality of meals, mainly in the younger age groups (18–24 and 25–34). Patients aged 18–24 indicated 37% more often the answer that they were only sometimes given tasty and fresh meals, and 14% less often that they were always served meals of adequate quality and hygienic standards. Patients aged 25–34 significantly more often (21%) indicated that their meals had never been tasty, fresh and hygienically served. A study on patient satisfaction with stationary health care services in the Kujawsko–Pomorskie Voivodship indicated a high level of patient dissatisfaction with the meals served. The respondents complained mainly about the temperature of the meals, the variety of dishes and the size of the meals served [26]. Similar results were obtained in another patient satisfaction study, where patients aged 65+ rated the meals much more highly [27]. 

Another important aspect of the quality of medical services is hospital accommodation, including the size of the rooms and the availability of separate diagnostics rooms that allow for the provision of medical services with respect for the patient’s privacy and dignity. In the study presented in this paper, the elderly patients assessed this aspect of health care much better than patients from other age groups. In the study, the patients drew attention, not only to the quality of services, but also to the conditions which they stayed in. The standard of equipment was viewed as an aspect that affected the patient’s comfort during hospitalization. The results of the study showed that the vast majority of patients were satisfied with the conditions which they stayed in [32,33,34]. A study on patient satisfaction with stationary health care in the Kujawsko–Pomorskie Voivodship showed that the conditions of hospital rooms, and the respect shown for patient’s dignity and privacy were assessed as bad and very bad by 6% of respondents [26].

In sum, providing patients with care that is safe, effective and responsive to patient needs is now recognized as the foremost objective of health systems in all OECD countries. To achieve this, it is necessary to measure the quality of care and to help managers in health entities identify the drivers of high-quality care as the cornerstones of quality improvement. Measuring patients’ opinions helps to evaluate aspects such as effectiveness and the achievement of desirable outcomes to ensure correct provision of evidence-based healthcare. Safety, reducing harm caused in the delivery of health care processes and patient-centered practice, placing the patient/user at the center of healthcare delivery, are critical [35].

### Limitations of the Study

A limitation of the study is the unrepresentativeness of the sample with respect to age, which does not allow conclusions to be drawn for the entire population. Half of the respondents were persons aged 25–44 years and only 10% of patients were over 65 years of age, which is the group that uses a large percentage of hospital services. Another limitation is that it was a one-off study. To constantly monitor the quality of health care and patient experience, and to introduce procedural improvements and improve the quality of services, continuous and regular research needs to be carried out.

## 5. Conclusions

Variables such as age and income had a statistically significant influence on the reported opinions of the respondents. Elderly patients highly valued most aspects of hospital care, such as treating patients with due care and respect, the quality and freshness of meals served in the hospital ward, effective pain treatment and respect for the patient’s privacy and dignity. 

There was a similar number of promoters (36%) and detractors (38%) of the quality of services. Detractors pointed mainly to the long waiting times for hospital admission to hospital, poor quality of medical and nursing care, and unappealing meals. The promoters reported a high quality of medical and nursing care as well as good accommodation conditions.

Regular studies on patient satisfaction with medical services in hospital wards provide information on patients’ opinions and, thus, are helpful in identifying areas of the operation of a medical entity that require improvement.

## Figures and Tables

**Table 1:** Analysis of patients’ recommendations of a hospital/hospital ward to family and friends.

Would You Recommend the Hospital Ward to Your Family and Friends?	NPS			
	All Respondents	Promoters	Neutral Respondents	Detractors
	%	%	%	%
0	4%	--	--	11%+
1	1%	--	--	2%+
2	4%	--	--	10%+
3	4%	--	--	11%+
4	7%	--	--	18%+
5	10%	--	--	26%+
6	8%	--	--	22%+
7	14%	--	52%+	--
8	13%	--	48%+	--
9	11%	30%+	--	--
10	25%	70%+	--	--

**Table 2:** Analysis of patients’ opinions on being treated with due interest and care by the medical staff.

How Often Were You Treated by Hospital Medical Staff with Due Interest and Care?	NPS				Age					
	All Respondents	Promoters	Neutral Respondents	Detractors	18–24	25–34	35–44	45–54	55–64	65+
	%	%	%	%	%	%	%	%	%	%
Never	5%	2%−	1%−	11%+	9%	6%	7%	--	1%−	1%−
Sometimes	26%	7%−	15%−	53%+	37%+	28%	26%	23%	18%−	19%
Usually	35%	26%−	58%+	26%−	37%	35%	31%	42%	33%	30%
Always	34%	65%+	26%−	10%−	17%−	30%	37%	35%	48%+	50%+

**Table 3:** Analysis of patients’ opinion on the quality and freshness of meals, and the hygiene of serving meals in hospital.

How Often during Your Hospital Stay Were the Meals Tasty, Fresh and Served Hygienically?	NPS				Age					
	All Respondents	Promoters	Neutral Respondents	Detractors	18–24	25–34	35–44	45–54	55–64	65+
	%	%	%	%	%	%	%	%	%	%
Never	16%	5%−	9%−	33%+	22%	21%+	17%	11%−	12%	5%−
Sometimes	29%	18%−	32%	37%+	37%+	23%−	31%	32%	23%	31%
Usually	31%	33%	38%+	23%−	27%	34%	28%	36%	28%	31%
Always	24%	43%+	22%	6%−	14%−	22%	24%	22%	37%+	33%

**Table 4:** Analysis of how often medical staff washed or disinfected their hands in the presence of the patient before performing medical activities or touching bed sheets, bed, cupboard or other personal belongings of the patient.

How Often Did the Medical Staff Wash or Disinfect Their Hands in Your Presence before Performing Medical Tests, Procedures, and Toileting Activities, or Touching Bed Sheets, Bed, Cupboard or Your Personal Belongings?	NPS				Age					
	All Respondents	Promoters	Neutral Respondents	Detractors	18–24	25–34	35–44	45–54	55–64	65+
	%	%	%	%	%	%	%	%	%	%
Never	7%	2%−	3%−	14%+	9%	5%	8%	6%	5%	6%
Sometimes	20%	10%−	19%	31%+	21%	24%	22%	17%	13%−	15%
Usually	30%	21%−	36%+	35%+	37%	30%	28%	30%	29%	29%
Always	43%	67%+	42%	20%−	33%−	41%	42%	47%	52%	50%

**Table 5:** Analysis of how often pain treatment in hospital was effective.

How Often Was Pain Treatment Effective during Your Hospital Stay?	NPS				Age					
	All Respondents	Promoters	Neutral Respondents	Detractors	18–24	25–34	35–44	45–54	55–64	65+
	%	%	%	%	%	%	%	%	%	%
Does not concern (no need)	8%	4%−	8%	11%+	11%	5%−	8%	5%	11%	10%
Never	3%	1%−	--	8%+	7%	2%	5%	2%	1%−	3%
Sometimes	18%	6%−	12%−	36%+	22%	23%+	18%	17%	12%−	10%−
Usually	34%	25%−	44%+	35%	34%	39%	34%	35%	23%−	33%
Always	37%	64%+	35%	11%−	27%−	31%−	36%	41%	53%+	45%

**Table 6:** Analysis of patients’ opinions on hospital conditions and accessibility of diagnostics and treatment with respect for patient’s dignity and intimacy.

Did Hospital Conditions, e.g., the Size of the Room, Separate Diagnostics Rooms, Allow for the Treatment with Respect for Your Dignity and Intimacy?	NPS				Age					
	All Respondents	Promoters	Neutral Respondents	Detractors	18–24	25–34	35–44	45–54	55–64	65+
	%	%	%	%	%	%	%	%	%	%
Definitely NOT {1}	7%	2%−	--	19%+	11%	10%	8%	5%	3%−	3%−
Rather NOT {2}	22%	11%−	15%−	37%+	24%	25%	24%	20%	13%−	15%
Rather YES {3}	44%	36%−	61%+	39%−	48%	39%	42%	46%	46%	49%
Definitely YES {4}	27%	51%+	24%	6%−	17%−	25%	27%	29%	38%+	34%
Bottom two boxes	29%	13%−	15%−	55%+	34%	35%+	32%	25%	16%−	18%−
Top two boxes	71%	87%+	85%+	45%−	66%	65%−	68%	75%	84%+	83%+

**Table 7:** Analysis of patients’ opinion on the achievement of the best possible treatment result during hospitalization.

Was the Best Possible Treatment RESULT Achieved during Your Hospital Stay?	NPS				Age					
	All Respondents	Promoters	Neutral Respondents	Detractors	18–24	25–34	35–44	45–54	55–64	65+
	%	%	%	%	%	%	%	%	%	%
Definitely NOT {1}	8%	1%−	2%−	18%+	9%	11%	7%	6%	2%−	8%
Rather NOT {2}	16%	6%−	8%−	34%+	25%+	14%	16%	18%	16%	11%
Rather YES {3}	48%	42%−	66%+	42%−	52%	51%	46%	47%	47%	45%
Definitely YES {4}	28%	52%+	24%	6%−	15%−	24%	32%	29%	35%	36%
Bottom two boxes	24%	7%−	10%−	52%+	34%+	25%	22%	24%	18%	19%
Top two boxes	76%	93%+	90%+	48%−	66%−	75%	78%	76%	82%	81%

## Data Availability

All data is available from the corresponding author.
